# Structural and Metabolic Changes in Pregnant Rat Uterine and Adipose Tissue Induced by a High-Fat High-Sugar Diet

**DOI:** 10.3390/biom15030446

**Published:** 2025-03-20

**Authors:** Dina Šišljagić, Senka Blažetić, Milorad Zjalić, Irena Labak, Vedrana Ivić, Kálmán Ferenc Szűcs, Róbert Gáspár, Eszter Ducza, Sandor G. Vari, Andrijana Muller, Marija Heffer

**Affiliations:** 1 Clinic for Obstetrics and Gynecology, Clinical Hospital Center Osijek, 31000 Osijek, Croatia; dinasisljagic99@gmail.com (D.Š.); andrijana.muller@gmail.com (A.M.); 2 Department of Biology, Josip Juraj Strossmayer University of Osijek, 31000 Osijek, Croatia; ilabak@biologija.unios.hr; 3 Department for Medical Biology and Genetics, Faculty of Medicine Osijek, Josip Juraj Strossmayer University of Osijek, 31000 Osijek, Croatia; mzjalic@mefos.hr (M.Z.); vedrana.ivic@mefos.hr (V.I.); mheffer@mefos.hr (M.H.); 4 School of Medicine, University of Zagreb, 10000 Zagreb, Croatia; 5 Hungary BiopharmacyDepartment of Pharmacology and Pharmacotherapy, Albert-Szent-Györgyi Medical School, University of Szeged, 6720 Szeged, Hungary; szucs.kalman@med.u-szeged.hu (K.F.S.); gaspar.robert@med.u-szeged.hu (R.G.); 6 Department of Pharmacodynamics and Biopharmacy, Faculty of Pharmacy, University of Szeged, 6720 Szeged, Hungary; ducza.eszter@szte.hu; 7 International Research and Innovation in Medicine Program, Cedars–Sinai Medical Center, Los Angeles, CA 90048, USA; sandor.vari@cshs.org; 8 Department of Obstetrics and Gynecology, Faculty of Medicine Osijek, J. J. Strossmayer University of Osijek, 31000 Osijek, Croatia

**Keywords:** myometrium, endometrium, UCP proteins, insulin receptor, leptin receptor

## Abstract

Pregnancy presents specific metabolic demands, and disruption caused by a high-fat high-sugar diet (HFHSD) have been associated with significant complications, including maternal health risk, fetal developmental issues, and infertility. Obesity-related changes in the uterine tissues may contribute to these challenges. This study analyzed structural changes in the uterus and adipose tissue of pregnant rats on gestation day 22 fed an HFHSD using various staining techniques. Hematoxylin and eosin staining showed morphological changes in the adipose tissue and the uterine structure, including the lumen size and the thickness of the myometrium, endometrium, and perimetrium. The amount of collagen in the uterus was determined by PicroSirius red staining, while PAS-D staining was used to observe glycogen content. Key protein expressions, such as insulin and leptin receptors and UCP1 and UCP3, were analyzed by immunohistochemistry. The HFHSD promoted hypertrophy of visceral and gonadal adipocytes, suggesting metabolic alterations. By the end of pregnancy, a significant reduction in uterine lumen size was observed. Additionally, a decrease in insulin and higher leptin receptor expressions in the myometrium indicated significant physiological alteration. These findings offer insight into how an HFHSD affects uterine structure and function during late pregnancy but should be interpreted within the physiological context of gestation-related metabolic changes. Further research is needed to understand the functional consequences of these alterations on reproductive and metabolic health.

## 1. Introduction

The high-fat and high-sugar diet (HFHSD) in modern societies has been an extremely important factor associated with the global epidemic of obesity. Such a diet is consistently linked to metabolic disorders [1], chronic inflammation [2], and systemic metabolic abnormalities that change the morphology and function of different organs [3,4,5]. Among the different systems that may be affected, the reproductive system is of specific importance due to its importance in health and disease [6]. The uterus is a dynamic structure that undergoes significant structural and functional changes during an individual’s life, influenced by different factors such as hormones [7], nutrients [8], and environmental conditions [9]. In the context of obesity, adipose tissue is one of the primary organs that undergoes changes and affects other systems [10]. In addition to being an energy reservoir, adipose tissue is an active endocrine organ. Despite its relevance in reproductive health and metabolic disorders, the interplay between diet-induced obesity, adipose tissue dynamics, and uterine function remains poorly understood.

Different studies have demonstrated that an HFHSD is one of the key factors leading to obesity, insulin resistance [11], and systemic inflammation [12,13] in animal models, making it a valuable tool for investigating the underlying mechanisms of diet-induced pathophysiology. In rats, such a diet leads to significant changes in uterine contractility and inflammatory processes [14], as well as the induction of insulin resistance in female rats [15], potentially disrupting reproductive processes and metabolic balance. The changes in the uterus, a vital reproductive organ, occur across its three distinct layers: the endometrium, myometrium, and perimetrium [16]. Together, these layers support essential reproductive functions such as implantation, gestation, and parturition [12]—energy-dependent processes that make the uterus a metabolically active organ. The effect of energy balance on uterine health is crucial to identify mechanisms underlying reproductive dysfunctions caused by metabolic disorders. According to previous research, obesity during pregnancy reduces the mass of the fetus and placenta [17] and leads to a redistribution of different types of adipose tissue, particularly from subcutaneous to visceral fat. This redistribution may influence pregnancy outcomes by altering intra-abdominal pressure and affecting maternal and fetal health. Maintaining a healthy and fully functional uterus is important for proper implantation of a fertilized egg, a normal pregnancy, and delivery [18,19,20]. Insulin and leptin signaling, collagen remodeling, glycogen storage, and mitochondrial energy regulation are key factors essential for understanding changes related to uterine health and fertility outcomes. However, the specific morpho-functional changes in the uterus resulting from such a diet remained underexplored. This study aims to analyze the impact of an HFHSD on adipose and uterine tissues of pregnant rats on gestation day 22, focusing on morphological and functional changes. By investigating the effects of this diet, we aim to clarify the mechanisms that link diet-induced obesity to uterine dysfunction during pregnancy. We hypothesize that an HFHSD during pregnancy induces structural and functional alterations in adipose and uterine tissues, resulting in changes in uterine morphology and protein expression patterns that may contribute to impaired uterine function.

## 2. Materials and Methods

### 2.1. Animal Model and Study Design

The study was conducted on adipose and uterine tissues of Sprague–Dawley rats obtained from Animalab Ltd. (Vác, Hungary) collected on the 22nd day of pregnancy as part of the project “REECOP-CMSC SENIOR SCIENTIST (RCSS) GRANT 2018-2020 #012”, “The role of obesity-induced low-grade inflammation in the adipokine signaling in pregnant rat uterus”.

**Figure 1:**
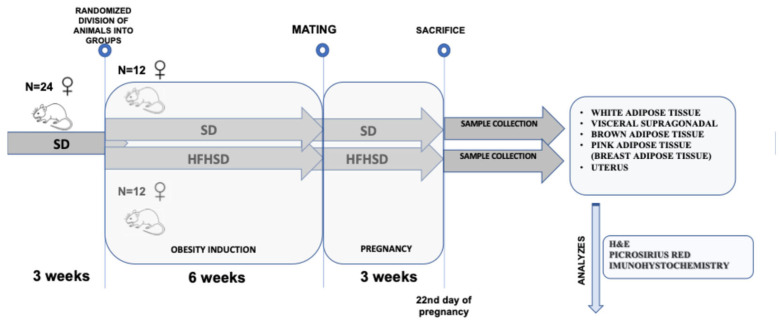
Schematic presentation of study design. Abbreviations: H&E—hematoxylin and eosin histological staining, HFHSD—rats on high-fat high-sugar diet, SD—rats on standard diet.

The first phase of the study was conducted entirely at the University of Szeged, Hungary. Three-week-old female rats were randomized in two groups: the control group (12 rats) fed a standard diet (SD) (1314, Altromin Spezialfutter GmbH & Co. KG, Lage, Germany) and the experimental group (HFHSD) fed an HFHSD (C1011, Altromin Spezialfutter GmbH & Co. KG, Lage, Germany) till 9 weeks of age (Figure 1). At 10 weeks of age, rats were mated with male Sprague–Dawley rats and kept on their assigned diet until the end of the pregnancy (22nd day). Adipose tissue and uterine samples were analyzed at the Laboratory for Neurobiology, Faculty of Medicine Osijek and in the Laboratory for Biochemistry at Department of Biology, J.J. Strossmayer University of Osijek, Croatia (Figure 1).

The study was approved by the National Scientific Ethics Committee on Animal Experimentation (registration number: IV./3071/2016., date 15 August 2016) and was conducted under the direction of Prof. Robert Gaspar, PharmD, PhD.

### 2.2. Sample Preparation

On the 22nd day of pregnancy, rats were sacrificed using isoflurane anesthesia. and Samples of the uterus and adipose tissue (visceral (VF)and gonadal (VF)) were collected, fixed in 4% paraformaldehyde for 16 h, and embedded in paraffin blocks. These blocks were then cut using a Leica RM 550 microtome (Leica, Vienna, Austria) at the Department of Histology and Embryology, Faculty of Medicine Osijek. Every 10th section (6 µm thick), from a total of 50 sections of each adipose tissue, was carefully selected and mounted on silane-coated glass slides to avoid multiple measurements of the same adipocyte. Uterus samples were collected as a 5 µm thick section, also every 10th section for a total of 15 sections per animal. The tissue sections prepared in this way were used for histological staining and immunohistochemical analysis. Digital micrographs (3 micrographs from each section) of the stained tissue were collected by an Olympus D70 camera (Olympus, Hamburg, Germany) set up on a Zeiss Axioskop 2 MOT microscope (Carl Zeiss Microscopy, Thornwood, NY, USA). Adipose tissue was analyzed in CellProfiler (v. 4.2.6), while the Image J program was used for all other analyses. The experiment was conducted in a blinded manner, ensuring that the researchers analyzing the samples were unaware of the group assignments to minimize potential bias.

### 2.3. Hematoxylin and Eosin Staining of Adipose Tissue and Uterus

To analyze the morphological changes of the visceral and gonadal adipose tissue (adipocyte number and area) and the uterus (uterine lumen and myometrium, endometrium, and perimetrium thickness), hematoxylin and eosin staining was performed. Paraffin-embedded tissue sections were first deparaffinized by immersion in xylene for 10 min, followed by rehydration through a sequential ethanol series—twice in 100%, then in 96%, and finally in 70% ethanol solutions for 5 min each. After ethanol treatment, the sections were washed in distilled water for 5 min. Staining was conducted in the following order: Mayer’s hematoxylin for 10 min, distilled water for 1 min, tap water for 10 min, and distilled water for 1 min. Eosin staining was performed with sequential eosin Y for 30 s; distilled water for 5 s; 70%, 96%, and 100% ethanol, 5 dips in each; 100% ethanol for 3 min; and xylene for 5 min, after which they were allowed to air-dry at room temperature, covered with mounting medium (Histomount, Histological Mount Medium, National Diagnostics, Cat. No.: HS-103) and coverslipped. This method was used for measuring the number and area of adipocytes in adipose tissue, and the diameters of the endometrium, myometrium, and perimetrium in the uterus as well as the area of the uterine lumen were assessed.

### 2.4. PicroSirius Red Staining of Uterus

PicroSirius red staining was used to evaluate the amount of collagen in the uterus. Paraffin-embedded sections were deparaffinized with xylene and rehydrated through a graded ethanol series (100%, 95%, 70%, and 50%) followed by rinsing in distilled water. Slides were then incubated in 0.1% Sirius red dissolved in saturated aqueous picric acid for 1 h at room temperature. After staining, slides were rinsed in 0.5% acetic acid to remove excess stain, dehydrated through an ascending ethanol series, and cleared with xylene, and mounting with a resin-based medium under a coverslip was performed.

### 2.5. Periodic Acid Schiff (PAS) Staining

Periodic acid Schiff with diastase (PAS-D) staining was used to analyze uterine glycogen content. Following deparaffinization and rehydration (see Section 2.3), sections were pretreated with diastase solution for 1 h at 37 °C and washed. The staining procedure included periodic acid (5 min), tap water (2 min), Schiff’s reagent in the dark (10 min), tap water (10 min), and modified Mayer’s hematoxylin (30 s), followed by a 2 min tap water rinse. Sections were then dehydrated in graded alcohol (70%, 80%, 95%, and 100%) for 2 min each, cleared in xylene, and mounted with DPX Mountant (Biognost Ltd., Zagreb, Croatia; Cat. No.: BM-500).

### 2.6. Uterus Immunohistochemistry

Uterine sections were deparaffinized and rehydrated (see Section 2.3). For epitope recovery, sections were treated in citrate buffer (pH = 6.0) in a water bath at 95 °C for 40 min, followed by cooling and washing in 1× PBS and distilled water. Immunohistochemistry was performed at 4 °C, beginning with pretreatment in 1% H_2_O_2_ for 30 min, followed by blocking in 5% goat serum and 1% BSA in 1× PBS for 2 h. Sections were than incubated with specific primary antibodies: mouse anti-ObR diluted 1:50 (Santa Cruz, SC, Dallas, TX, SAD, Cat. No.: 8391); rabbit anti-IR diluted 1:250 (IR-α; Santa Cruz Biotechnology, Dallas, TX, USA; Cat. No.: SC-710); rabbit anti-UCP1 diluted 1:1000 (Abcam, Cat. No.: ab10983); and rabbit anti-UCP3 diluted 1:3000 (Invitrogen, Cat. No.: PA1-24895). Next, sections were washed three times in 1x PBS (10 min each) and incubated in secondary antibodies for 4 h. Biotinylated goat anti-mouse IgG diluted 1:1000 (Santa Cruz, SC, Dallas, TX, USA, Cat. No.: SC-2039) for ObR and biotinylated goat anti-rabbit IgG diluted 1:1000 (Jackson ImmunoResearch Laboratories, Inc. West Grove, PA, USA; Cat. No.: 115-067-003) was followed by washing three times in 1× PBS 3 (10 min each). The secondary antibody was detected using the Vectastain ABC kit (Vector Laboratories Inc., Burlingame, CA, USA) through a 1 h incubation. The sections were washed in 1× PBS and visualized using the peroxidase substrate kit (DAB) (Vector Laboratories Inc., Burlingame, CA, USA). Subsequently, the sections were mounted on slides, air-dried, and coverslipped with Vectamount (Vector Laboratories Inc., Newark, United States).

### 2.7. Statistical Analysis

Statistical analysis was performed using Statistica 13 software (TIBCO, Palo Alto, CA, USA), with *p* < 0.05 as the statistical significance level.

Between-group comparisons to determine the influence of an HFHSD on adipocyte morphology were calculated using an independent samples *t*-test, assuming normal distribution, after performing the Shapiro–Wilk test for normality and Levene’s test for homogeneity of variances. The Mann–Whitney U Test was used to analyze the difference in uterus morphological changes, while ANOVA, followed by Bonferroni post hoc test was performed for all uterine receptors.

## 3. Results

Data on the fundamental effects of the HFHSD in pregnant rats, such as food consumption, body weight, organ weights, and glucose tolerance, were previously published in a study on uterine contractility and cervical resistance [14]. This approach aligns with the 3R principles by maximizing the value of existing data and minimizing the need for additional animal use.

### 3.1. Morphological Changes of Adipose Tissue and Uterus

**Figure 2:**
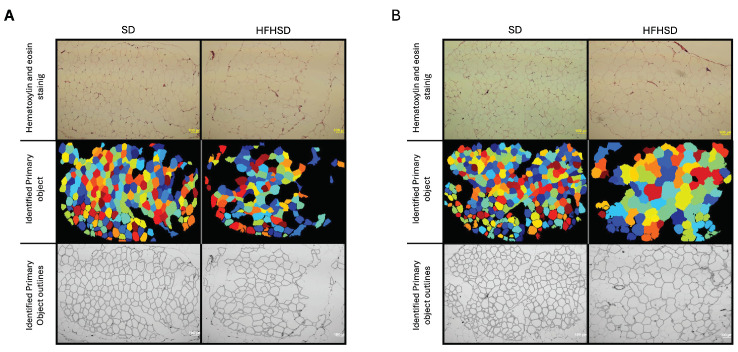
Hematoxylin-eosin staining (upper row) and output images from Cell Profiler (middle row—identified objects; lower row—outlines of identified objects) of gonadal (**A**) and visceral (**B**) adipose tissue. Abbreviations: GF—gonadal fat, HFHSD—high-fat high-sugar diet, SD—standard diet, VF—visceral fat. Scale: 100 μm.

**Figure 3:**
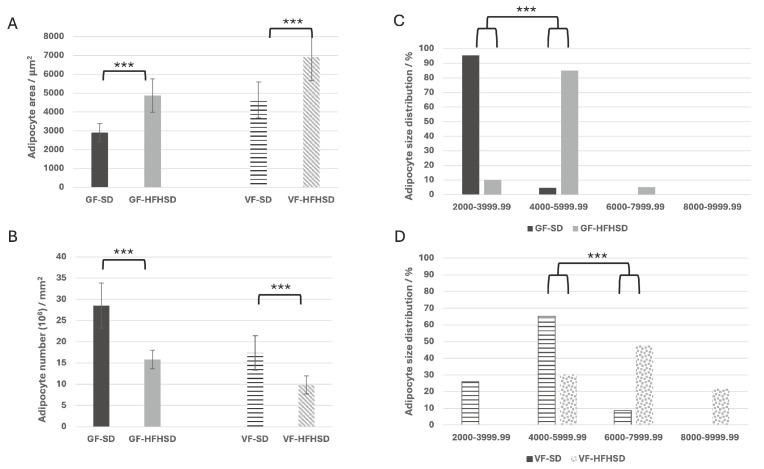
Adipocyte cell area (**A**), number (**B**), and size distribution - classes (**C**,**D**). Abbreviations: GF—gonadal fat, HFHSD—high-fat high-sugar diet, SD—standard diet, VF—visceral fat, *** *p* ≤ 0.001. Data represent average results of 10 animals per group. Error bars indicate standard deviation (SD).

Sprague–Dawley rats fed an HFHSD during pregnancy showed a statistically significant increase in the adipocyte surface area of both gonadal (*p* < 0.001) and visceral (*p* < 0.001) adipose tissue. In GF, the mean adipocyte surface area increased from 2911.63 μm^2^ in the standard diet group (GF-SD) to 4872.12 μm^2^ in the HFHSD group (GF-HFHSD). Similarly, for visceral adipose tissue, the mean surface area increased from 4640.34 μm^2^ (VF-SD) to 6917.06 μm^2^ (VF-HFHSD) (Figure 2 and Figure 3A). The increase in adipocyte size was accompanied by a significant reduction in adipocyte number per unit area (*p* < 0.001). In GF, the count dropped from 28.5 in GF-SD to 15.8 in the GF-HFHSD group. Similarly, the VF showed a decrease from 17.37 (VF-SD) to 9.83 in the VF-HFHSD group (Figure 3B).

The distribution of adipocyte surface areas in GF and VF was further analyzed by categorizing adipocytes into six classes based on their surface area (class 1 (<2000 µm^2^); class 2 (2000–3999.99 µm^2^); class 3 (4000–5999.9 µm^2^); class 4 (6000–7999.9 µm^2^); class 5 (8000–9999.99 µm^2^); and class 6 (>10,000 µm^2^). An HFHSD significantly altered the distribution of adipocyte classes in both GF and VF. In GF, 95.5% is classified as class 2, while in VF, most adipocytes (65.21%) belong to class 3. Under HFHSD conditions, there was a statistically significant shift toward larger adipocytes. In GF, 85% of adipocytes belonged to class 3, whereas in VF, 47.82% of adipocytes were classified as class 4 (*p* < 0.001), with 30.43% still falling into class 3. Notably, in the SD group, neither GF nor VF contained adipocytes from class 5. In contrast, in the VF of the HFHSD group (VF-HFHSD), class 5 adipocytes accounted for 21.73% of the total.

**Figure 4:**
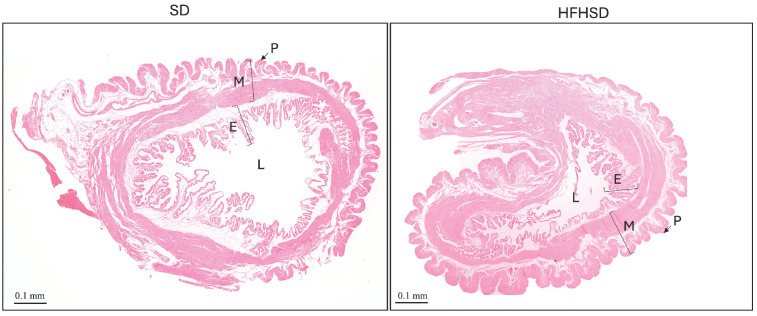
Histological analysis of rat uterus cross section stained by hematoxylin and eosin, magnification 5×. Abbreviations: E—endometrium, HFHSD—high-fat high-sugar diet, L—uterine lumen, M—myometrium, P—perimetrium, SD—standard diet.

**Figure 5:**
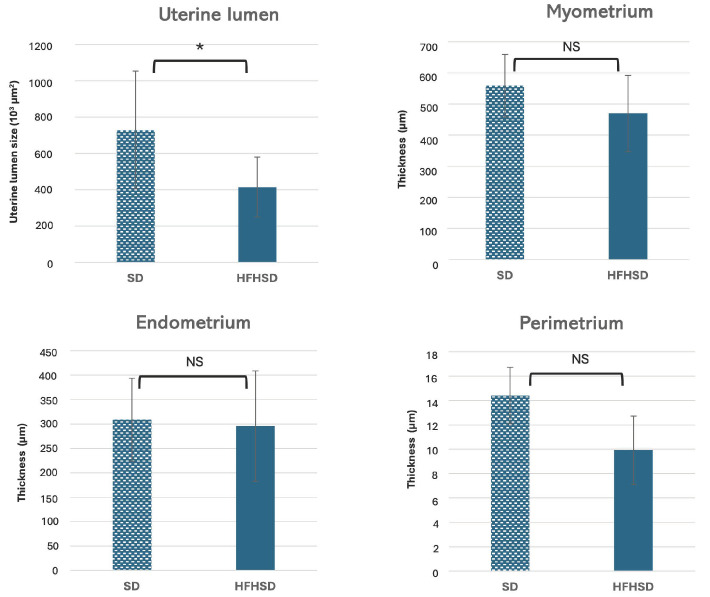
Size of rat uterine lumen and thickness of myometrium, endometrium and perimetrium. Abbreviations: HFHSD—high-fat high-sugar diet, SD—standard diet; * *p* ≤ 0.05, NS—non-significant. Data represent average results of 10 animals per group. Error bars indicate standard deviation (SD).

Histological analysis of rat uterus included measurement of the uterus lumen and thickness of the myometrium, endometrium, and perimetrium. Uterine lumen has been significantly reduced in the HFHSD group (*p* = 0.005), while there was no significant difference in the thickness of the myometrium, endometrium, and perimetrium (Figure 4 and Figure 5).

### 3.2. Glycogen Deposition and Collagen in Uterus

To gain insight into the metabolic activity of the uterus, glycogen was analyzed as a crucial source of glucose, particularly during early pregnancy. Collagen was evaluated as a key structural component supporting uterine growth and strength necessary to sustain pregnancy, a process that is highly energy-dependent. The results indicated no statistically significant differences in glycogen or collagen levels as a result of an HFHSD (Supplementary Datas S1 and S2).

### 3.3. Insulin and Leptin Receptors Expression in Uterus

**Figure 6:**
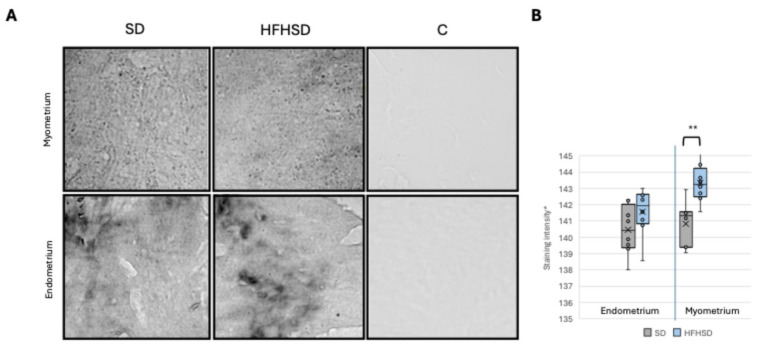
Expression of IR in rat myometrium (upper row) and endometrium (lower row). Representative immunohistochemistry microscopic images, magnification 40× (**A**). Relative immunoreactive staining intensity of IR in rat uterus (**B**). Data represent average results of 10 animals per group. Error bars indicate standard deviation. * 0 = greatest intensity; 255 = no staining. Abbreviations: C—control, HFHSD—high-fat high-sugar diet, SD—standard diet. ** *p* < 0.01.

**Figure 7:**
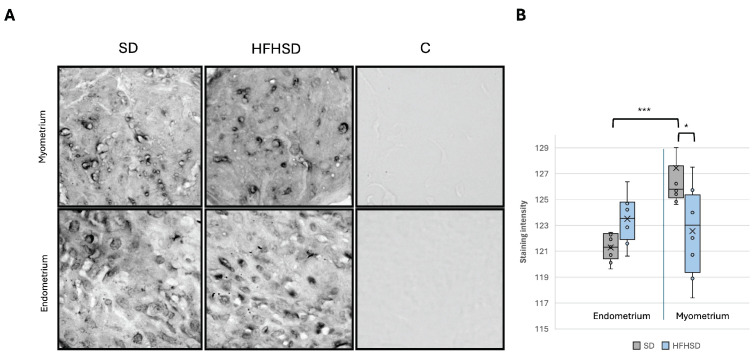
Expression of ObR in rat myometrium (upper row) and endometrium (lower row). Representative immunohistochemistry microscopic images, magnification 40× (**A**). Relative immunoreactive staining intensity of ObR in rat uterus (**B**). Data represent average results of 10 animals per group. Error bars indicate standard deviation. *0 = greatest intensity; 255 = no staining. Abbreviations: C—control, HFHSD—high-fat high-sugar diet, SD—standard diet. * *p* < 0.05; *** *p* ≤ 0.001.

To provide insights into how metabolic signals influence uterine physiology, particularly in the context of metabolic disorders and diet-induced changes, the expression of insulin (IR) and leptin receptors (ObR) was analyzed in the myometrium and endometrium of the uterus. The results showed a significantly decreased IR expression in the myometrium (*p* = 0.007) of the HFHSD group, while no significant change was observed in the endometrium compared to the SD group (Figure 6). In contrast, the expression of ObR in the myometrium of the HFHSD group was significantly increased (*p* = 0.019) compared to the SD group. Additionally, in the endometrium of rats in the SD group, ObR expression was significantly higher compared to the myometrium of the same group (*p* = 0.001) (Figure 7).

### 3.4. Uncoupling Protein 1 and 3 Expression in Uterus

**Figure 8:**
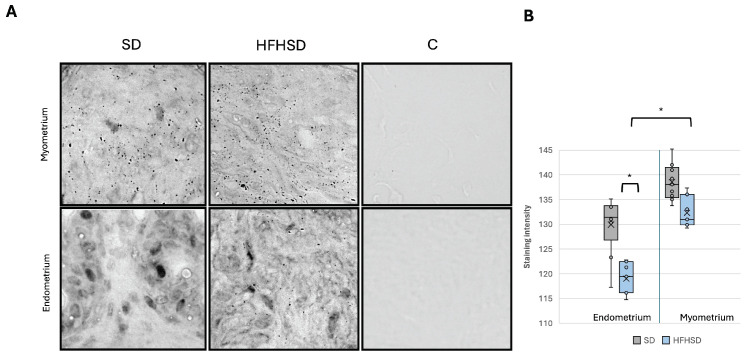
Expression of UCP3 in rat myometrium (upper row) and endometrium (lower row). Representative immunohistochemistry microscopic images, magnification 40× (**A**). Relative immunoreactive staining intensity of UCP3 in rat uterus (**B**). Data represent average results of 10 animals per group. Error bars indicate standard deviation. 0 = greatest intensity; 255 = no staining. Abbreviations: C—control, HFHSD—high-fat high-sugar diet, SD—standard diet. * *p* < 0.05.

The mitochondrial uncoupling proteins (UCPs) expression in the uterus was analyzed to evaluate uterine energy expenditure and thermogenesis. Although there was a visible trend toward decreased UCP1 expression in response to an HFHSD, this difference was not significant (Supplementary Data S3). In contrast, UCP3 expression was significantly increased in the endometrium of rats from the HFHSD group (*p* = 0.036). Furthermore, the HFHSD group exhibited significantly higher UCP3 expression in the endometrium compared to the myometrium (*p* = 0.021) (Figure 8).

The table with the key results related to adipose tissue, uterine morphology, and various receptor and protein expressions in the HFHSD and SD groups is presented as Supplementary Data S4.

## 4. Discussion

Structural and metabolic changes in the uterus and adipose tissue in rats fed an HFHSD at the end of pregnancy (gestation day 22) were analyzed in this study with the emphasis on energy metabolism-related biomarkers. Pregnancy introduces unique metabolic demands on adipose tissue to meet the energy needs of both the mother and developing fetus. Our results demonstrated significant alterations in both adipose tissue and uterine morphology. The changes in adipocyte size and distribution show significant metabolic alterations brought about by the HFHSD, especially in GF and VF, which are both important sites for energy storage and endocrine signals during pregnancy [21,22]. The shift toward larger adipocyte classes in both GF and VF demonstrates adipocyte hypertrophy, the main cause of obesity and generally related to metabolic and cardiovascular disease [23,24]. Those results are in accordance with other studies that report that an HFHSD mostly leads to adipose tissue expansion through hypertrophy rather than hyperplasia [15,25]. The significant increase in VF, which is more metabolically active than other types of fat, in the HFHSD group indicates potential systemic metabolic changes, including altered insulin signaling and inflammatory responses [26,27]. In women, an increased amount of VF positively correlates with the development of uterine fibroids [28]. The hypertrophy of adipocytes was accompanied by a statistically significant decrease in the number of adipocytes per unit area in both visceral and gonadal adipocytes. This reduction suggests that lipid storage has shifted toward accommodating larger lipid droplets within fewer adipocytes, which could provoke metabolic stress leading to metabolic dysfunction [29]. Under an HFHSD, the shift toward larger adipocyte classes in both GF and VF reflects an overall increase in lipid storage capacity which generally could contribute to gestational insulin resistance [30]. Different studies indicated a relationship between adipose tissue function and uterine health. Impaired function of adipose tissue, as seen in obesity or metabolic disorders, can negatively affect uterine health and contribute to pregnancy complications, like impaired implantation, fetal growth restriction, or preterm birth []. In this study, structural changes in the uterus—uterine lumen size, as well as myometrium, endometrium, and perimetrium thickness—were analyzed to highlight the effect of an HFHSD. A significant reduction in uterine lumen size was observed in the HFHSD group. The uterine lumen has an important role in supporting proper placental development [31], and the observed reduction suggests potential disruptions in these processes which are essential for successful pregnancy outcomes. These results suggest that while the overall uterine structure maintains flexibility in terms of layer thickness, the decreased uterine lumen size may still reflect early signs of compromised uterine function or compensatory mechanisms aimed at maintaining uterine integrity under metabolic stress. The absence of significant changes in glycogen and collagen levels in this study still does not exclude the possibility of other HFHSD-induced effects on uterine physiology. To further investigate potential functional disruptions in uterine metabolism and structure associated with this dietary pattern, we analyzed the expression of IR and ObR. The observed significant decrease in IR expression in the myometrium of the HFHSD group suggests possible alterations in insulin signaling within uterine smooth muscle. This reduction may affect uterine contractility and glucose utilization, as diet-induced insulin resistance is typically associated with downregulated IR expression [32]. Notably, the lack of change in IR expression in the endometrium may indicate a uterine-specific response to metabolic disruption, possibly reflecting differential metabolic requirements or compensatory mechanisms within the endometrium to retain its important roles in implantation and placental development. Glycogen synthesis is promoted by the binding of insulin to the IR on endometrial epithelial cells [33]; the lack of IR expression difference may be consistent with the absence of changes in glycogen storage. In contrast to IR, ObR expression was significantly increased in the myometrium, highlighting the complex interaction between leptin signaling and uterine physiology. Previous studies suggest that leptin, an adipokine important for energy homeostasis and reproduction, affects uterine contractility and inflammatory responses [34,35]. Additionally, the increased ObR expression observed in the endometrium of the SD group compared to the myometrium underlines the tissue-specific regulatory functions of leptin, which are critical for endometrial receptivity and early pregnancy events. Srinivasan et. al (2021) reported that leptin inhibits mouse uterine contraction by stimulating short forms of the ObR [36], and results from our animal model indicate a reduction in uterine contractility in the HFHSD group [14]. These findings align with the cross downregulation phenomenon of IR and ObR in the uterus, as previously reported [37]. Pregnancy requires substantial amounts of energy for endometrial remodeling, placental development, and uterine contractility, all of which are essential for a successful heathy pregnancy. Dysregulation of either insulin or leptin signaling may compromise mitochondrial efficiency and alter the expression of uncoupling proteins (UCPs) in the uterus, potentially leading to metabolic stress [38,39]. The increased UCP3 expression observed in the endometrium of the HFHSD group suggests enhanced mitochondrial activity or altered energy demands in this tissue. UCP3 is known to modulate oxidative stress and facilitate fatty acid metabolism [38,40], potentially representing a compensatory response to the metabolic changes induced by an HFHSD. This finding aligns with reports of increased UCP3 expression in other tissues exposed to a high-fat diet [41,42], reflecting an adaptive response to lipid overload. Although UCP1 is primarily involved in thermogenesis [43], its variable expression in the uterus suggests a minimal role in uterine energy homeostasis regulation. UCP1 is predominantly expressed in brown adipose tissue [44], and its low activity in the non-adipose tissues, such as the uterus, may explain the observed findings. Alternatively, uterine energy needs may be regulated through pathways independent of UCP1-mediated thermogenesis, particularly during pregnancy when glucose consumption is crucial for fetal development. Although the exact functional significance of UCPs in the uterus remains unclear, their presence suggests s critical role in maintaining energy efficiency and cellular adaptations to the increased metabolic demands of pregnancy.

Given that late pregnancy itself induces significant metabolic and inflammatory changes, the interpretation of our findings must consider this physiological context. While our histological and immunohistochemical analyses provide valuable insights into tissue morphology and semi-quantitative protein expression, they cannot be used for direct conclusions about systemic metabolic dysregulation, insulin resistance, or inflammation. Future studies incorporating functional assessments, such as implantation success rates or direct metabolic profiling, would be necessary to determine the precise impact of an HFHS diet on reproductive outcomes.

### 4.1. The Strengths and Limitations of the Study

The strength of this study lies in its detailed histological and immunohistochemical assessments of both adipose and uterine tissue, providing insights into the potential effects of diet on reproductive health. The use of rat models is appropriate as they offer control over dietary conditions and allow for the dissection of specific metabolic pathways and biomarkers. Additionally, the study avoids unnecessary duplication of animal use by referencing previously published data on food consumption, body weight, organ weights, and glucose tolerance. However, one of the main limitations of this study is that it was conducted in an animal model, and results from rats do not always directly translate to human physiology. While rat models provide invaluable insights into metabolic processes, further studies in human or clinical models are necessary to validate the observed effects. Moreover, the absence of functional assays, such as implantation success rates, fetal growth outcomes, or metabolic profiling, limits the ability to make direct conclusions about the reproductive success or the systemic metabolic dysregulation caused by an HFHS diet.

### 4.2. The Potential Clinical Implication and Future Research

The findings of this study highlight the potential clinical implications of HFHS diets during pregnancy, particularly in contributing to gestational insulin resistance, altered uterine function, and compromised placental development. These effects may increase the risk of complications like preterm birth, fetal growth restriction, and impaired implantation. Future research should focus on functional assessments such as implantation success and fetal development, alongside exploring the role of inflammation and epigenetic changes in response to high-fat diets. Additionally, clinical studies in human populations are necessary to confirm these findings and evaluate their broader impact on maternal and fetal health outcomes.

## 5. Conclusions

This study aimed to outline the effects of an HFHSD on uterine physiology and maternal health. Structural and molecular alternations were characterized by adipocyte hypertrophy, a reduced uterine lumen size, changes in IR and ObR expression in myometrium, and increased levels of UCP3 in the endometrium. While no significant changes were observed in glycogen and collagen levels, these findings suggest potential shifts in uterine function and signaling pathways.

## Data Availability

The original contributions presented in this study are included in the article/supplementary material. Further inquiries can be directed to the corresponding author.

## Supplementary Materials

The following supporting information can be downloaded at https://www.mdpi.com/article/10.3390/biom15030446/s1, Supplementary Data S1. Collagen deposition. (A) Representative Picrosirius Red staining microscopic and analysed images, magnification 5×. (B) Percentage of collagen deposition in rat uterus. Abbreviations: SD—standard diet; HFHSD—high fat high sugar diet; NS—non-significant. Supplementary Data S2. Periodic acid-Schiff (PAS) staining analysis of rat uterus. (A) Representative PAS-stained microscopic images, magnification 5× (B) Glycogen deposition in the rat uterus. Abbreviations: SD — standard diet; HFHSD—high-fat high-sugar diet; NS—non-significant. Supplementary Data S3. Expression of UCP1 in rat myometrium (upper row) and endometrium (lower row). Representative immunohistochemistry microscopic images, magnification 40× (A). Relative immunoreactive staining intensity of UCP3 in rat uterus (B). Data represent average results of 10 animals per group. Error bars indicate standard deviation. 0 = greatest intensity; 255 = no staining. Abbreviations: C—control, HFHSD—high-fat high-sugar diet, SD—standard diet. Supplementary Data S4. The key results related to adipose tissue, uterine morphology, and various receptor and protein expressions in the HFHS and SD diet groups.

## Abbreviations

The following abbreviations are used in this manuscript:

GF	Gonadal fat
HFHSD	High-fat high-sugar diet
IR	Insulin receptor
ObR	Leptin receptor
SD	Standard diet
UCP1	Uncoupling protein 1
UCP3	Uncoupling protein 3
VF	Visceral fat
