# Supplementary material for: Structural and Metabolic Changes in Pregnant Rat Uterine and Adipose Tissue Induced by a High-Fat High-Sugar Diet
Source: Biomolecules. 2025 Mar 20;15(3):446. doi: 10.3390/biom15030446 (PMC11940457; doi:10.3390/biom15030446)
Supplement: Supplementary file 1 [file biomolecules-15-00446-s001.zip › biomolecules-3474043-supplementary.pdf]

Supplementary Data S1. Collagen deposition. (A) Representative Picrosirius Red staining microscopic and analysed images, magnification 5x. (B) Percentage of collagen deposition in rat uterus. Abbreviations: SD - standard diet; HFHSD – high fat high sugar diet; NS—non-significant.

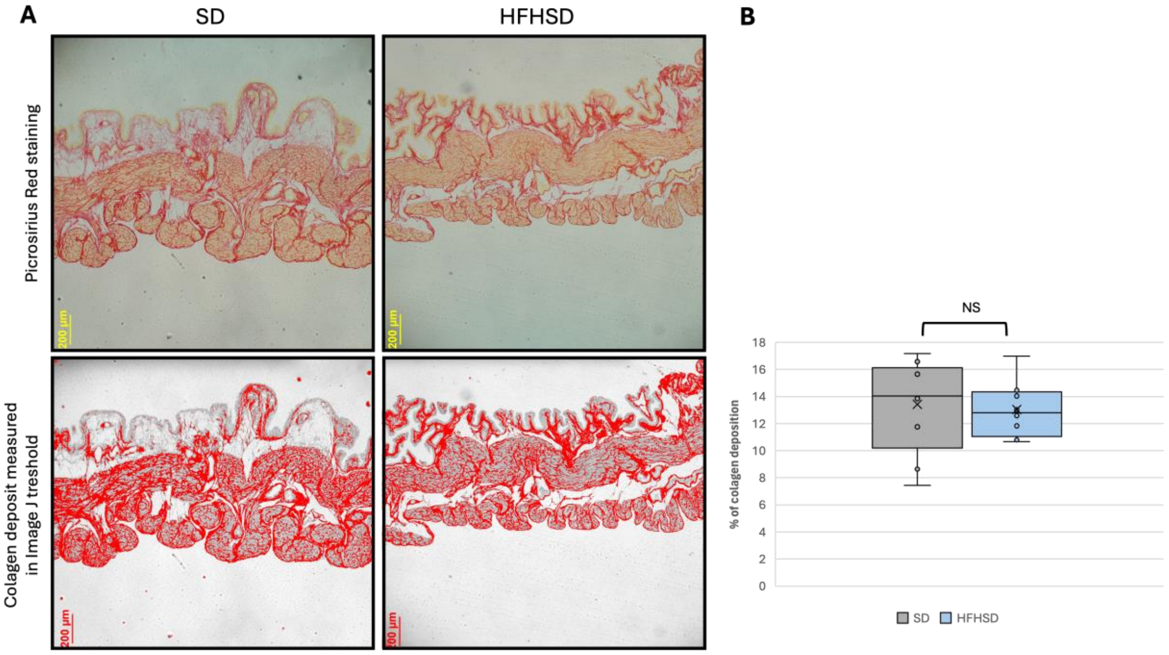

Supplementary Data S2. Periodic acid-Schiff (PAS) staining analysis of rat uterus. (A) Representative PAS-stained microscopic images, magnification 5x (B) Glycogen deposition in the rat uterus. Abbreviations: SD — standard diet; HFHSD — high-fat high-sugar diet; NS — non-significant.

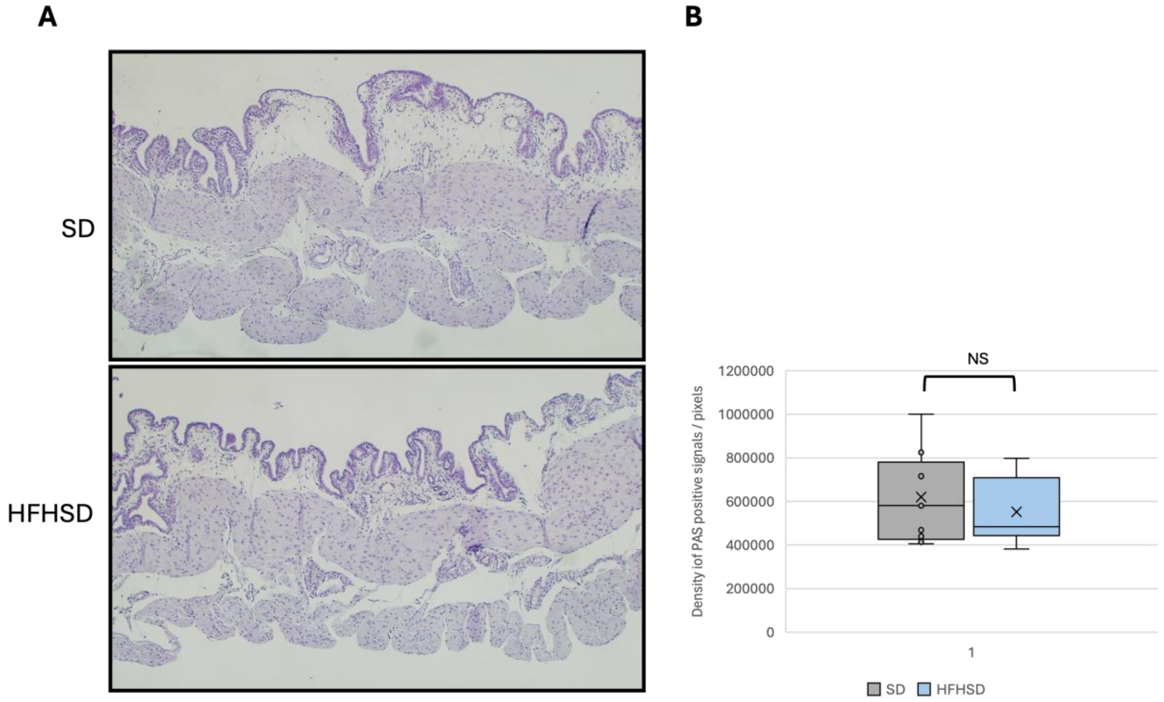

Supplementary Data S3. Expression of UCP1 in rat myometrium (upper row) and endometrium (lower row). Representative immunohistochemistry microscopic images, magnification 40× (A). Relative immunoreactive staining intensity of UCP3 in rat uterus (B). Data represent average results of 10 animals per group. Error bars indicate standard deviation. 0 = greatest intensity; 255 = no staining. Abbreviations: C—control, HFHSD—high-fat high-sugar diet, SD—standard diet.

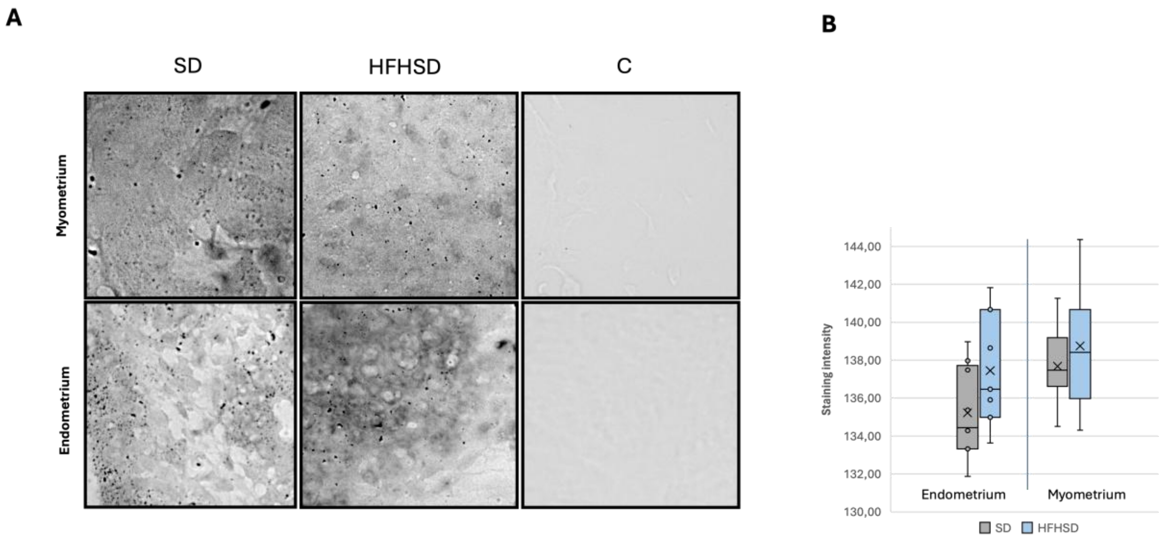

Supplementary Data S4. The key results related to adipose tissue, uterine morphology, and various receptor and protein expressions in the HFHS and SD diet groups.

Parameter	HFHSD vs. SD group
**Adipose Tissue (Gonadal and Visceral)**	-Significant increase in adipocyte surface area (p < 0.001)
	- Decrease in number of adipocytes per unit area (p < 0.001)
	- Shift towards larger adipocytes, especially in visceral fat (class 5 adipocytes: 21.73%)
**Uterine Lumen**	- Significant decrease in uterine lumen size (p = 0.005)
**Uterine Myometrium, Endometrium, Perimetrium**	- No significant changes in thickness of myometrium, endometrium, or perimetrium
**Glycogen Levels in Uterus**	- No significant difference in glycogen levels
**Collagen Levels in Uterus**	- No significant difference in collagen levels
**Insulin Receptor (IR) Expression**	- Significant decrease in IR expression in myometrium (p = 0.007)
**Leptin Receptor (ObR) Expression**	- Significant increase in ObR expression in myometrium (p = 0.019)
	- Higher ObR expression in endometrium compared to myometrium (p = 0.001)
**Uncoupling Protein 1 (UCP1) Expression**	- Trend towards decreased UCP1 expression, but not statistically significant
**Uncoupling Protein 3 (UCP3) Expression**	- Significant increase in UCP3 expression in endometrium (p = 0.036)
	- Higher UCP3 expression in endometrium compared to myometrium (p = 0.021)
